# Head/neck paragangliomas: focus on tumor location, mutational status and plasma methoxytyramine

**DOI:** 10.1530/ERC-21-0359

**Published:** 2022-02-16

**Authors:** Susan Richter, Bei Qiu, Mirthe Ghering, Carola Kunath, Georgiana Constantinescu, Charlotte Luths, Christina Pamporaki, Nicole Bechmann, Leah Meuter, Aleksandra Kwapiszewska, Timo Deutschbein, Svenja Nölting, Mirko Peitzsch, Mercedes Robledo, Aleksander Prejbisz, Karel Pacak, Volker Gudziol, Henri J L M Timmers, Graeme Eisenhofer

**Affiliations:** 1 Institute for Clinical Chemistry and Laboratory Medicine, University Hospital Carl Gustav Carus, Medical Faculty Carl Gustav Carus, Technische Universität Dresden, Dresden, Germany; 2 Department of Internal Medicine, Radboud University Medical Centre, Nijmegen, The Netherlands; 3 Department of Medicine III, University Hospital Carl Gustav Carus, Medical Faculty Carl Gustav Carus, Technische Universität Dresden, Dresden, Germany; 4 Eunice Kennedy Shriver National Institute of Child Health and Human Development, National Institutes of Health, Bethesda, Maryland, USA; 5 Department of Hypertension, Institute of Cardiology, Warsaw, Poland; 6 Division of Endocrinology and Diabetes, Department of Internal Medicine I, University Hospital, University of Würzburg, Würzburg, Germany; 7 Medicover Oldenburg MVZ, Oldenburg, Germany; 8 Medizinische Klinik and Poliklinik IV, Ludwig-Maximilians-Universität München, Munich, Germany; 9 Department for Endocrinology, Diabetology and Clinical Nutrition, UniversitätsSpital Zürich, Zurich, Switzerland; 10 Hereditary Endocrine Cancer Group, CNIO, Madrid, Spain; 11 Centro de Investigación Biomédica en Red de Enfermedades Raras (CIBERER), Madrid, Spain; 12 Department of Otorhinolaryngology, University Hospital Carl Gustav Carus, Technische Universität Dresden, Dresden, Germany; 13 Klinik für Hals-Nasen-Ohrenheilkunde, Kopf- und Hals-Chirurgie, Plastische Operationen, Städtisches Klinikum Dresden, Akademisches Lehrkrankenhaus der Technischen Universität Dresden, Dresden, Germany

**Keywords:** biochemical phenotype, methoxytyramine, normetanephrine, sex-related differences, succinate dehydrogenase mutations, tumor size

## Abstract

Head and neck paragangliomas (HNPGLs) are tumors of parasympathetic origin that occur at variable locations and are often secondary to germline mutations in succinate dehydrogenase (SDH) subunit genes. Occasionally, these tumors produce catecholamines. Here, we assessed whether different locations of HNPGLs relate to the presence of *SDHx* mutations, catecholamine production and other presentations. In this multicenter study, we collected clinical and biochemical data from 244 patients with HNPGLs and 71 patients without HNPGLs. We clarified that jugulotympanic HNPGLs have distinct features. In particular, 88% of jugulotympanic HNPGLs arose in women, among whom only 24% occurred due to *SDHx* mutations compared to 55% in men. Jugulotympanic HNPGLs were also rarely bilateral, were of a smaller size and were less often metastatic compared to carotid body and vagal HNPGLs. Furthermore, we showed that plasma concentrations of methoxytyramine (MTY) were higher (*P*  < 0.0001) in patients with HNPGL than without HNPGL, whereas plasma normetanephrine did not differ. Only 3.7% of patients showed strong increases in plasma normetanephrine. Plasma MTY was positively related to tumor size but did not relate to the presence of *SDHx* mutations or tumor location. Our findings confirm that increases in plasma MTY represent the main catecholamine-related biochemical feature of patients with HNPGLs. We expect that more sensitive analytical methods will make biochemical testing of HNPGLs more practical in the future and enable more than the current 30% of patients to be identified with dopamine-producing HNPGLs. The sex-dependent differences in the development of HNPGLs may have relevance to the diagnosis, management and outcomes of these tumors.

## Introduction

Head and neck paragangliomas (HNPGLs) arise from parasympathetic ganglia within the skull base or upper neck. They are highly vascularized but slowly growing tumors that metastatize in 6–13% of cases (Jansen *et al.* 2000, Lee *et al.* 2002, Papaspyrou *et al.* 2012, Mediouni *et al.* 2014). In contrast to other paragangliomas, symptoms due to HNPGLs are often related to local mass effects rather than catecholamine secretion; some are clinically silent and discovered incidentally during imaging studies (Taieb *et al.* 2014, Cass *et al.* 2020). It is well established that germline mutations in succinate dehydrogenase (*SDHx*) genes predispose to pheochromocytoma and paraganglioma (Amar *et al.* 2005, Benn *et al.* 2006, Turkova *et al.* 2016). Syndromic presentations of HNPGLs are associated with mutations in all *SDHx* genes and the assembly factor *SDHAF2* (Guha *et al.* 2019). Additionally, epigenetic inactivation of *SDHC* via promoter methylation has been described in one HNPGL (Bernardo-Castineira *et al.* 2018). Rare cases of HNPGLs in patients with Von Hippel–Lindau syndrome or multiple endocrine neoplasia type 2 have been reported; however, due to lack of somatic mutation testing, their circumstantial association in patients with these syndromes cannot be excluded (Boedeker *et al.* 2009).

In 60% of cases, HNPGLs are located at the carotid bifurcation and present as carotid body paragangliomas, often without symptoms (Cass *et al.* 2020). The most common tumors of the middle ear are tympanic paragangliomas, which may present with symptoms of pulsatile tinnitus, hearing loss or vertigo. In close proximity, HNPGLs from the jugular foramen can arise and cause similar symptoms as tympanic tumors. When lesions extend from the jugular foramen into the tympanic cavity, they are referred to as jugulotympanic paragangliomas. These three groups together account for about 30% of HNPGLs. The rarest group with 5–10% of cases arises along the vagus nerve, commonly at the inferior ganglion (ganglion nodosum). They are mostly asymptomatic, but large tumors can cause dysphagia, hoarseness and vocal cord paralysis, similar to large carotid body HNPGLs.

Biochemical screening to detect HNPGLs is of limited value, since these tumors rarely produce catecholamines (Erickson *et al.* 2001, van Duinen *et al.* 2013, Smith *et al.* 2021). Evidence from these studies suggests that HNPGLs may produce dopamine, which is best assessed from measurements of plasma methoxytyramine (MTY); however, only the study of van Duinen (2013) included measurements of plasma MTY but did not include an appropriate comparison group to assess any significance of observed increases in plasma MTY. So far, no study has examined any relationships of catecholamine biochemical phenotypes of HNPGLs to genotype or specific tumoral locations. Apart from underlying differences in catecholamine biosynthetic machinery, it is unknown why some tumors are biochemically active and others are not.

In the present study, we hypothesized that highly variable biochemical presentations of HNPGLs may depend on both the location and size of the tumors and presence of underlying *SDHx* mutations. The study also allowed for the evaluation of relationships of different HNPGL locations with other phenotypic features and presentations of patients. Using data from 244 European and USA patients with HNPGLs, we analyzed the presence of *SDHx* mutations and plasma concentrations of catecholamine O-methylated metabolites MTY, normetanephrine (NMN) and metanephrine (MN) in relation to tumor locations. To establish the clinical relevance of plasma metabolites in patients with HNPGL, we included a cohort of 71 patients in whom HNPGL was excluded and compared increases of urinary and plasma metabolites in a subset of patients with HNPGLs in whom these measurements were available.

## Patients and methods

### Patients

**Figure 1:**
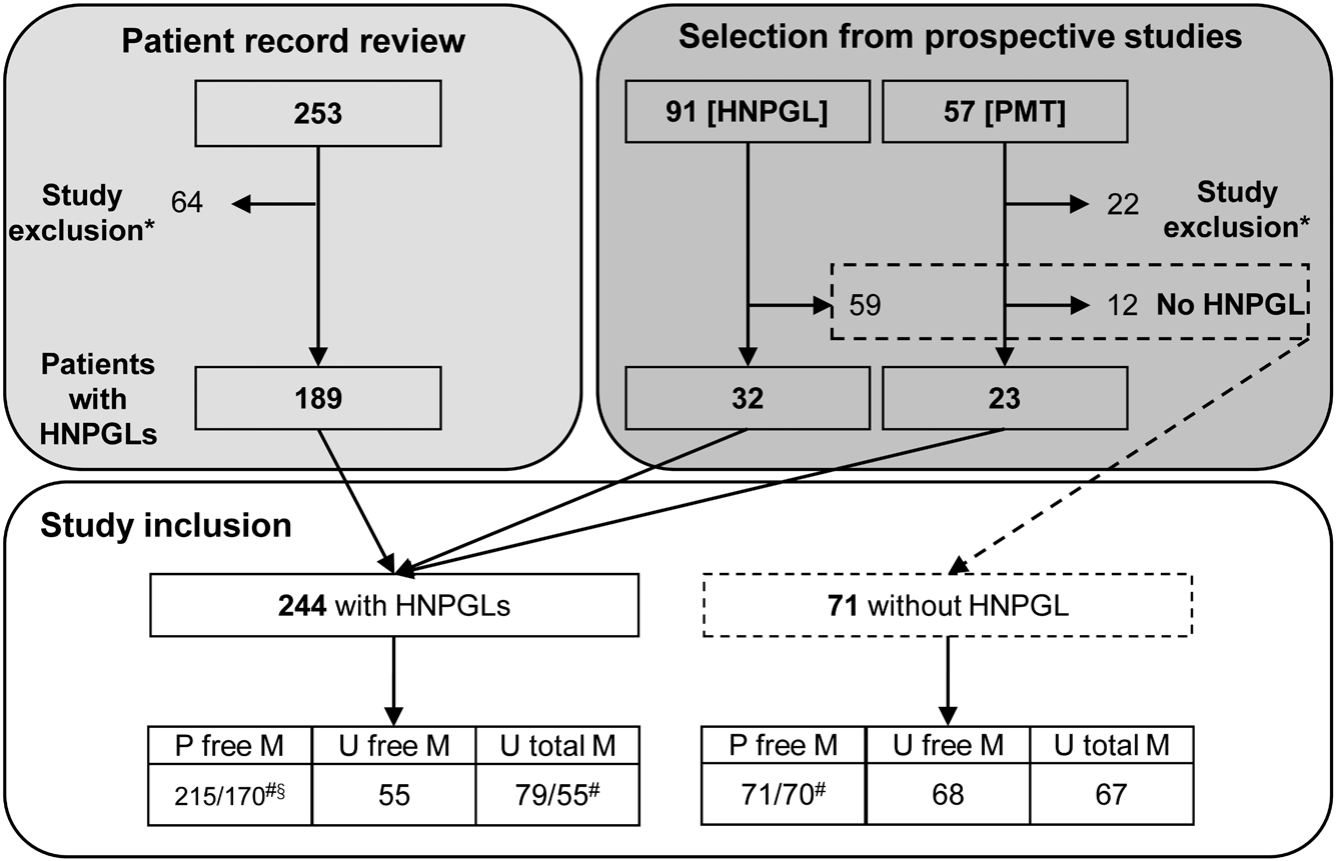
Patient inclusion. Patients with HNPGLs were included retrospectively from two centers based on patient record review and from two prospective studies coordinated in Dresden. The HNPGL study included Dresden patients with suspicion for HNPGL, whereas prospective monoamine-producing tumor study (PMT) is a multicenter study of biochemical profiles of monoamine-producing tumors. *Study exclusion was based on incomplete records, objection against data use, and other pheochromocytomas/paragangliomas or metastases present besides HNPGLs; #Biochemistry for MTY available for fewer patients than for NMN and MN; §51 measurements for plasma MTY (Nijmegen) were provided as lower limit of detection <100 pmol/L and excluded in some analysis, totaling to 119 patients with plasma MTY. P, plasma; U, urine; M, metanephrines and MTY.

This observational study included an initial population of 401 patients from centers in Germany (Dresden, Munich and Würzburg), the Netherlands (Nijmegen), Poland (Warsaw), as well as the NIH in Bethesda, USA. Patients were included via two different routes: (1) according to two prospective studies based at Dresden and (2) from a retrospective review of patient records at Nijmegen and the NIH (Fig. 1).

The two studies at Dresden included the multicenter prospective monoamine-producing tumor (PMT) study, which involved the enrolment of patients between September 2010 and October 2019 and a dedicated prospective HNPGL screening study based at the Department of Otorhinolaryngology in Dresden, which involved the enrolment of patients between January 2013 and April 2018. The PMT study included patients with suspicion or risk of pheochromocytoma or paraganglioma based on clinical findings or known germline mutations of tumor susceptibility genes (details at https://pmt-study.pressor.org/). The HNPGL screening study, on the other hand, included patients with imaging findings of a mass in the head and neck region.

Inclusion of patients with HNPGLs from a retrospective review of patient records involved patients with confirmed HNPGLs between January 2000 and June 2020 in Nijmegen and between June 1999 and January 2015 at the NIH. All patients included via studies based on Dresden or the NIH provided signed informed consent under protocols approved by local ethics committees, namely the Ethikkommission an der Technischen Universität Dresden, the Ethikkommission der Ludwig-Maximilians-Universität München, the Ethik-Kommission der Universität Würzburg, the Local Ethics Committee at the National Institute of Cardiology (Warsaw) and the NIH Institutional Review Board. Patients from Nijmegen signed a general consent not specific to this study protocol; for retrospective inclusion of patients, a waiver was obtained from the Medical Ethics Review Committee based on considerations that the study was not subject to the Medical Research Involving Human Subjects Act so that specific patient agreement was not required.

Exclusion criteria were incomplete records, objections against data use and the presence of pheochromocytoma or sympathetic paragangliomas that could contribute to increase in plasma or urinary catecholamine metabolites independently of HNPGLs. Similarly, the presence of metastases simultaneously with HNPGLs and for which the source of metastases could not be determined provided an additional basis for study exclusion. On this basis, 64 of the 253 patients from the retrospective cohort and 22 of the 148 patients from the prospective cohort were excluded from the final study population (Fig. 1).

That final population included 244 patients with HNPGLs and 71 patients in whom HNPGLs and catecholamine-producing tumors had been excluded. The latter patients were included to enable diagnostic comparison of biochemical test results between patients with and without HNPGL. All patients without disease had a clinical need for diagnostic screening for HNPGL, and either had a head and neck tumor for which an HNPGL was excluded based on the pathological review (59 patients from the HNPGL screening study) or had a known hereditary condition predisposing to HNPGLs (12 patients from the PMT study) with HNPGL and catecholamine-producing tumors excluded by patient follow-up as detailed previously (Eisenhofer *et al.* 2018).

### Clinical diagnosis and presentation

Diagnosis of HNPGLs was based on histopathology of resected or biopsied tumors. This followed imaging studies such as sonography, MRI, CT and at some centers functional imaging with radiolabeled somatostatin analogs. Imaging studies were either carried out as part of routine periodic surveillance due to a genetic predisposition to HNPGLs (e.g. an *SDHx* mutation) or a clinical presentation that led to suspicion of a head and neck tumor. Such presentations included a noticeable swelling or finding of palpable mass in the skull base, with or without other signs and symptoms of tumors. Such signs and symptoms included difficulties with swallowing, pulsations in the ear and other cranial nerve deficits. In some patients, catecholamine-related signs and symptoms were recorded, such as hypertension, headache, palpitations, excess sweatiness, nausea, dizziness, vomiting and fatigue. For patients who did not undergo surgery or biopsy, a diagnosis of HNPGL was achieved by functional imaging, such as with PET combined with CT and employing (^68^Ga)Ga-DOTA-TATE as the radiolabeled imaging agent.

### HNPGL locations

Locations of HNPGLs were based on imaging data. For the purposes of data analyses, specific locations were established according to four groups: (1) carotid body, (2) jugulotympanic, which included tympanic and jugular HNPGLs, (3) vagal and (4) multifocal, according to the presence of multiple simultaneous HNPGLs at different locations. Eight patients were presented with solitary HNPGLs located in parapharyngeal or paratracheal locations, in the tongue, the cervical ganglion, the thyroid or low in the neck. These cases were excluded from the analyses that focused on the four main head and neck locations as outlined above.

### Genetic testing

Germline mutations in established susceptibility genes were evaluated by centers of origin or by the Centro Nacional de Investigaciones Oncológicas (CNIO, Madrid, Spain) through a collaborative multicenter study (PMT study) using Sanger sequencing and/or next-generation sequencing, and multiplex ligation-dependent probe amplification or custom array comparative genomic hybridization for deletion detection. Variants detected in *SDHx* genes met the standards of the American College of Medical Genetics and Genomics and the Association for Molecular Pathology (Richards *et al.* 2015).

### Biochemical testing

In all centers except Nijmegen, blood samples were drawn in the morning following an overnight fast and at least 20 min of supine rest. In Nijmegen, supine sampling was performed for 35% of patients. Plasma O-methylated catecholamine metabolites, including MN, NMN and MTY, as well as 24-h urinary-free and total fractionated metanephrines and MTY, were measured by either liquid chromatography with tandem mass spectrometry (LC-MS/MS) or HPLC with electrochemical detection (LC-ECD). For all patients enrolled at Dresden and some patients enrolled at Nijmegen (41%), measurements of plasma and urinary metabolites were performed by LC-MS/MS (Peitzsch *et al.* 2013*a*, *b*, 2015). Comparability of results by LC-ECD and LC-MS/MS assays of plasma metabolites was previously established (Peitzsch *et al.* 2013*b*).

### Statistics

Statistical analyses were performed with the software package JMP Pro 15. Wilcoxon test and Steel–Dwass all pairs were used for non-parametric comparisons of numeric data in two and multiple groups, respectively. Fisher’s exact test plus analysis of means for proportions was performed for categorical data. Proportions of true- and false-negative results, as well as true- and false-positive results (i.e. sensitivity and specificity), were first established using previously defined cut-offs for metanephrines and MTY optimized for patients with chromaffin cell tumors (Eisenhofer *et al.* 2019). Thereafter, as described in the ‘Results’ section, cut-offs for plasma MTY were further optimized to establish the best performance for patients with HNPGLs using receiver operating characteristic (ROC) curve analysis and the derived Youden index. For logistic regression analysis, numeric values were transformed logarithmically before data input.

## Results

### Study population

As outlined in Fig. 1, after exclusions, the final study population comprised 244 patients with and 71 patients without HNPGLs. More women than men presented with HNPGLs, while more male patients were included in the group without HNPGLs (Table 1). Age was distributed similarly between groups. Germline *SDHx* mutations were detected in 55% of HNPGL patients. HNPGLs associated with *SDHA*, *SDHAF2*, *SDHB*, *SDHC* and *SDHD* mutations were present in 9, 12, 17, 9 and 85 patients, respectively. Genetic variants are listed in Table 2.

**Table 1:** Characteristics of patients with and without HNPGLs.

	HNPGL patients	Patients w/o HNPGL
*n*	244	71
Sex, females (%)	169 (69)	29 (41)
Age (at biochemistry)^a^	52 (11–89) years	56 (19–86) years
Germline *SDHx* mutations (%)	132^b^ (55.9)	10^c^
Germline *VHL* mutations	0^b^	2^c^

^a^Displayed is median (range); ^b^236 of 244 patients were genetically tested; ^c^12 patients of 71 were genetically tested as part of the PMT study.

**Table 2:** *SDHx*variants in our cohort (*n* = 132).

Gene	Variant	Cases with HNPGL	Cases with metastases
SDHA	c.91C>T, p.(Arg31*)	5	0
*NM_004168.4*	c.553_554insA, p.(Ala186fs)	1	1
	c.778G>A, p.(Gly260Arg)	1	0
	c.1753C>T, p.(Arg585Trp)	2	0
Total		9	1
SDHAF2	c.232G>A, p.(Gly78Arg)	7	0
*NM_017841.4*	Unknown^a^	5	0
Total		12	0
SDHB	c.211A>G, p.(Met71Val)	1	0
*NM_003000.3*	c.268C>T, p.(Arg90*)	1	0
	c.412G>A, p.(Asp138Asn)	1	0
	c.418G>T, p.(Val140Phe)	2	0
	c.530G>A., p.(Arg177His)	2	0
	c.649C>T, p.(Arg217Cys)	2	1
	c.686_725del, p.(Glu229fs)	1	0
	c.725G>A, p.(Arg242His)	1	0
	c.806delT, p.(Met269fs)	1	0
	Large deletion	3	0
	Unknown^a^	2	0
Total		17	1
SDHC	c.179G>T, p.(Ser60Ile)	3	1
*NM_003001.5*	c.214C>T (p.Arg72Cys)	1	0
	c.218G>A, p.(Gly73Asp)	1	0
	c.379C>G, p.(His127Asp)	1	0
	c.397C>T, p.(Arg133*)	1	0
	Large deletion	1	0
	Unknown^a^	1	0
Total		9	1
SDHD	c.33C>A, p.(Cys11*)	14	3
*NM_003002.4*	c.42delinsTC, p.(Gly15fs)	1	0
	c.49C>T, p.(Arg17*)	2	1
	c.122dup, p.(Glu42fs)	1	0
	c.169+1G>A	1	0
	c.169+5G>T	1	0
	c.170-1G>T	1	0
	c.239T>G, p.(Leu80Arg)	1	0
	c.242C>T, p.(Pro81Leu)	4	0
	c.274G>T, p.(Asp92Tyr)	25	2
	c.284T>C, p.(Leu95Pro)	11	0
	c.341A>G, p.(Tyr114Cys)	2	0
	c.383T>C, p.(Leu128Pro)	4	1
	c.416T>C, p.(Leu139Pro)	3	0
	Large deletion	3	0
	Unknown^a^	11	0
Total		85	7

^a^Patients in whom close family members were confirmed to have a pathogenic mutation in the gene, but testing was not performed in this individual or patients from older records with missing information concerning the exact gene variant.

### Biochemical phenotype of HNPGL patients

**Figure 2:**
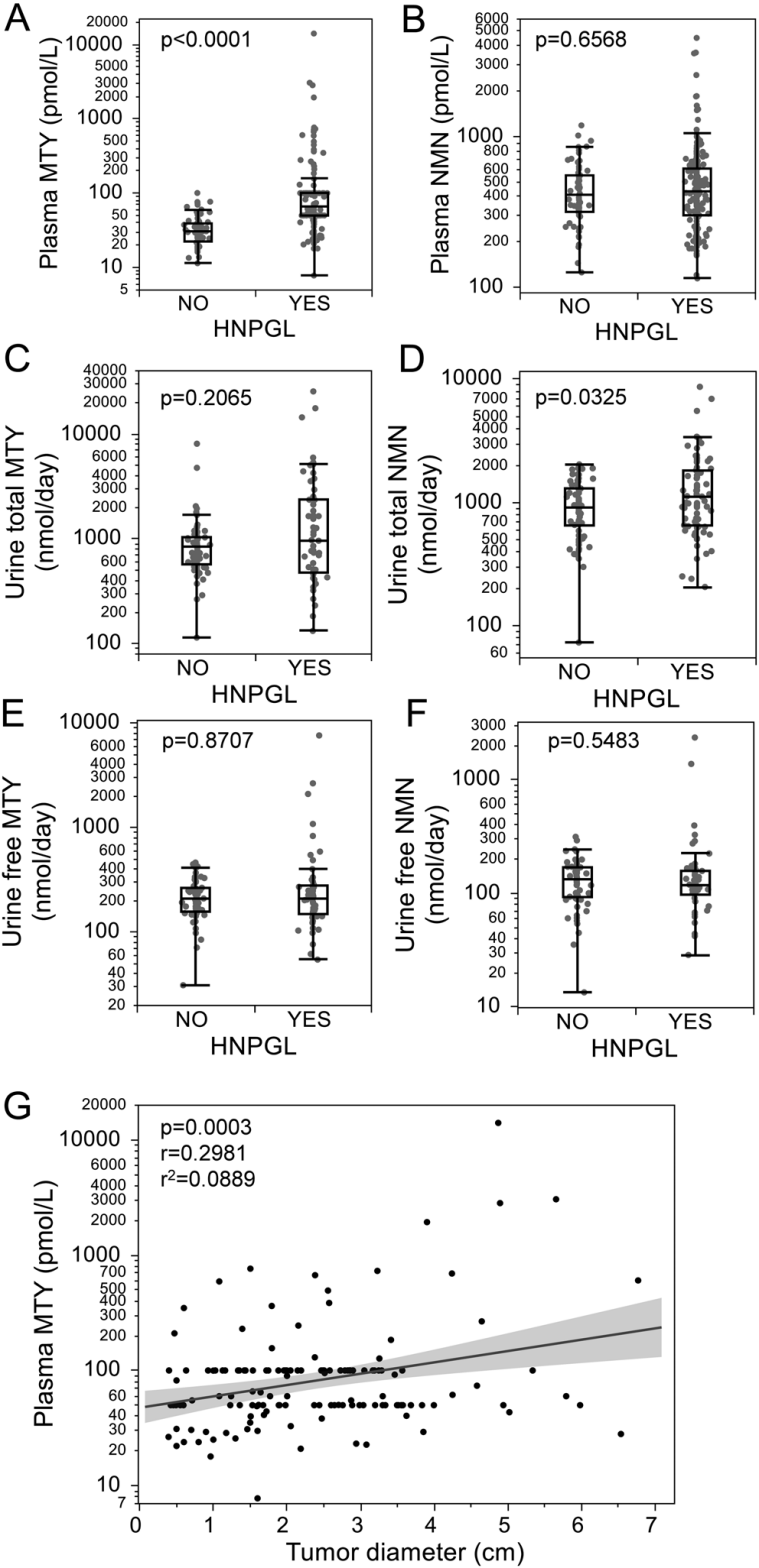
Plasma and urinary methoxytyramine (MTY) and normetanephrine (NMN) in patients with and without HNPGLs. (A, B, C, D, E and F) Significance was calculated by Wilcoxon rank-sum test. (G) Plasma MTY was plotted against tumor diameter, and linear regression with 95% CI was fitted.

Plasma concentrations of free MTY and urinary outputs of total (i.e. deconjugated) NMN were respectively 91% (*P*  < 0.0001) and 50% (*P* = 0.0325) higher in patients with than without HNPGLs, whereas plasma concentrations of free NMN, as well as urinary outputs of total MTY, free MTY and free NMN, showed no significant differences between patients with and without HNPGL (Fig. 2A, B, C, D, E and F). Examination of plasma concentrations of MTY and NMN according to HNPGL location revealed no significant differences, except for lower plasma concentrations of NMN in carotid body vs multiple and jugulotympanic HNPGLs (Table 3). Similarly, mutations in the *SDHx* genes were weakly associated with lower plasma NMN (Table 4). Using multivariate linear regression by standard least-squares including age, sex, *SDHx* mutational status, tumor volume and location, age has been identified as the most significant model parameter (*P* = 0.0009) and CB location as the second most significant contributor (*P* = 0.0187). *SDHx*status, on the other hand, was not significant. In contrast, plasma MTY was only correlated (*P* = 0.0003) with tumor diameter (Fig. 2G) and not with any other examined parameters. Urine total MTY and MN were somewhat higher in patients with *SDHx* vs non-*SDHx*-mutated HNPGLs; however, significance was marginal (Table 4). Similarly, there was a weak difference in urine total MN between patients with jugulotympanic and multiple HNPGLs (Table 3).

**Table 3:** HNPGL patient and tumor characteristics in respect to HNPGL location.

	Location				*P*^a^
	Jugulotympanic	Carotid body	Vagal	Multiple	
*n*	99	82	16	39	
Age at first diagnosis (years)	54.5 (11–89)^b,c^	42 (10–85)	51 (19–78)	36 (14–71)	<0.0001
Age at biochemistry (years)	57 (16–89)^b^	46 (11–85)	59.5 (25–78)	45.5 (21–76)	<0.0001
Sex (females, %)	87.9 (95%: 80.0–92.9)^d^	62.2 (95%: 51.4–72.0)	50.0 (95%: 30.0–66.3)	46.2 (95%: 31.6–61.4)^e^	<0.0001
Biochemistry					
Plasma MTY (pmol/L)	50 (18–671)	50 (8–14055)	50 (21–1937)	50 (40–731)	0.4102
Plasma NMN (pmol/L)	449 (114–3554)	365 (120–1840)^ c,f^	587 (173–1107)	502 (194–1850)	0.0039
Plasma MN (pmol/L)	161 (39–388)	138 (25–351)	149 (72–289)	164 (71–369)	0.1681
Urine total MTY (nmol/day)	523 (133–5953)	1022 (184–25454)	335 (323–347)	1648 (974–5135)	0.0128
Urine total NMN (nmol/day)	1170 (253–2889)	958 (242–5536)	1251 (207–3040)	1747 (568–3420)	0.2726
Urine total MN (nmol/day)	351 (93–1070)^c^	506 (87–1265)	370 (148–751)	683 (331–1380)^f^	0.0185
Genetics					
*SDHx* mutation (%)	27.4 (95%: 19.4–37.1)^d^	70.9 (95%: 60.1–79.7^e^	66.7 (95%: 41.7–84.8)	94.9 (95%: 83.1–98.6)^d^	<0.0001
*SDHx* mutation in females (%)	23.8 (95%: 16.0–33.9)^d^	66.0 (95%: 52.2–77.6)^e^	50.0 (95%: 21.5–78.5)	94.4 (95%: 74.2–99.0)^d^	<0.0001
*SDHx* mutation in males (%)	54.5 (95%: 28.0–78.7)	79.3 (95%: 61.6–90.2)	85.7 (95%: 48.7–97.4)	95.2 (95%: 77.3–99.2)	0.0484
Tumor characteristics					
Tumor volume (cm3)	1.6 (0.01–19.4)^b, c, g^	10.5 (0.03–162.2)	11.8 (0.1–111.9)	16.0 (1.4–79.5)	<0.0001
Tumor diameter (cm)	1.5 (0.3–3.3)^b, c, g^	2.7 (0.4–6.8)	2.8 (0.6–6.0)	3.1 (1.4–5.3)	<0.0001
Bilateral (%)	0^d^	29.3 (95%: 20.5–39.9)^e^	0	59.0 (95%: 43.4–72.9)^d^	<0.0001
Metastatic (%)	2.0 (95%: 0.6–7.1)^e^	11.0 (95%: 5.9–19.6)	6.3 (95%: 1.1–28.3)	12.8 (95%: 5.6–26.7)	0.0579

Data for proportions (%) are displayed as means (95% CI), whereas continuous data are displayed as medians (range). Missing data: for age at first diagnosis 1 JT; for age at biochemistry 8 JT, 4 CB, 2 vagal, 3 multiple; for plasma MTY 56 JT, 36 CB, 8 vagal, 25 multiple; for plasma NMN & MN 15 JT, 5 CB, 2 vagal, 7 multiple; for urine total MTY 79 JT, 60 CB, 14 vagal, 33 multiple; for urine total NMN & MN 70 JT, 52 CB, 12 vagal, 28 multiple; for SDHx mutation 4 JT, 3 CB, 1 vagal; for tumor volume/diameter 27 JT, 8 CB, 1 vagal, 7 multiple; for metastatic 1 JT.

^a^Pearson’s chi-squared or rank sums test; Steel Dwass test for multiple non-parametric comparisons: ^b^significant vs CB, ^c^significant vs multiple, ^f^significant vs vagal, ^g^significant vs JT; for categorical data analysis of means for proportion:^d^ *P*  < 0.001, ^e^ *P* < 0.05.

JT, jugulotympanic; CB, carotid body.

**Table 4:** HNPGL patient and tumor characteristics in respect to *SDHx* mutational status.

	SDHx		*P*^a^
	MUT	WT	
*n*	132	104	
Age at first diagnosis (years)	39 (10–78)	56 (11–89)	<0.0001
Age at biochemistry (years)	45 (11–78)	58.5 (16–89)	<0.0001
Sex (females, %)	57.6 (95%: 49.0–66.7)	85.6 (95%: 77.6–91.1)	<0.0001
Biochemistry			
Plasma MTY (pmol/L)	50 (8–14,055)	50 (18–3057)	0.1873
Plasma NMN (pmol/L)	422 (120–3554)	456 (114–4503)	0.0511
Plasma MN (pmol/L)	146 (25–378)	151 (39–366)	0.6264
Urine total MTY (nmol/day)	1601 (184–17,580)	689 (133–25,454)	0.0671
Urine total NMN (nmol/day)	1210 (242–5536)	1001 (207–8678)	0.5012
Urine total MN (nmol/day)	532 (87–1380)	351 (120–852)	0.0471
Tumor characteristics			
Tumor volume (cm^3^)	8.8 (0.03–162.2)	2.8 (0.01–111.9)	0.0006
Tumor diameter (cm)	2.6 (0.4–6.8)	1.7 (0.3–6.0)	0.0006
Bilateral (%)	33.3 (95%: 25.9–41.7)	1.9 (95%: 0.5–6.7)	<0.0001
Metastatic (%)	7.6 (95%: 4.2–13.4)	7.8 (95%: 4.0–14.6)	0.9564

Continuous data are displayed as medians (range), whereas data for proportions are displayed as means (95% CI). Missing data: for age at first diagnosis 1 WT; for age at biochemistry 6 MUT, 8 WT; for plasma MTY 71 MUT, 48 WT; for plasma NMN & MN 13 WT 13 MUT; for urine total MTY 100 MUT, 80 WT; for urine total NMN & MN 82 MUT, 77 WT; for tumor volume/diameter 18 MUT, 25 WT; for metastatic 1 WT.

^a^Pearson’s chi-squared or rank sums test.

MTY, methoxytyramine; MUT, mutant; NMN, normetanephrine.

### Distinct features of jugulotympanic tumors

Initial data analyses were performed with jugular and tympanic HNPGLs as separate groups; however, both entities showed similar features and subsequent statistical analyses combined these groups. Patients with jugulotympanic HNPGLs were diagnosed later in life than patients with carotid body or multiple tumors (Table 3). Jugulotympanic HNPGLs occurred significantly more often in women, while vagal tumors arose at equal proportions in men and women. Multiple HNPGLs were slightly more common in males, and carotid body tumors were somewhat more common in females; however, statistical significance was not reached.

**Figure 3:**
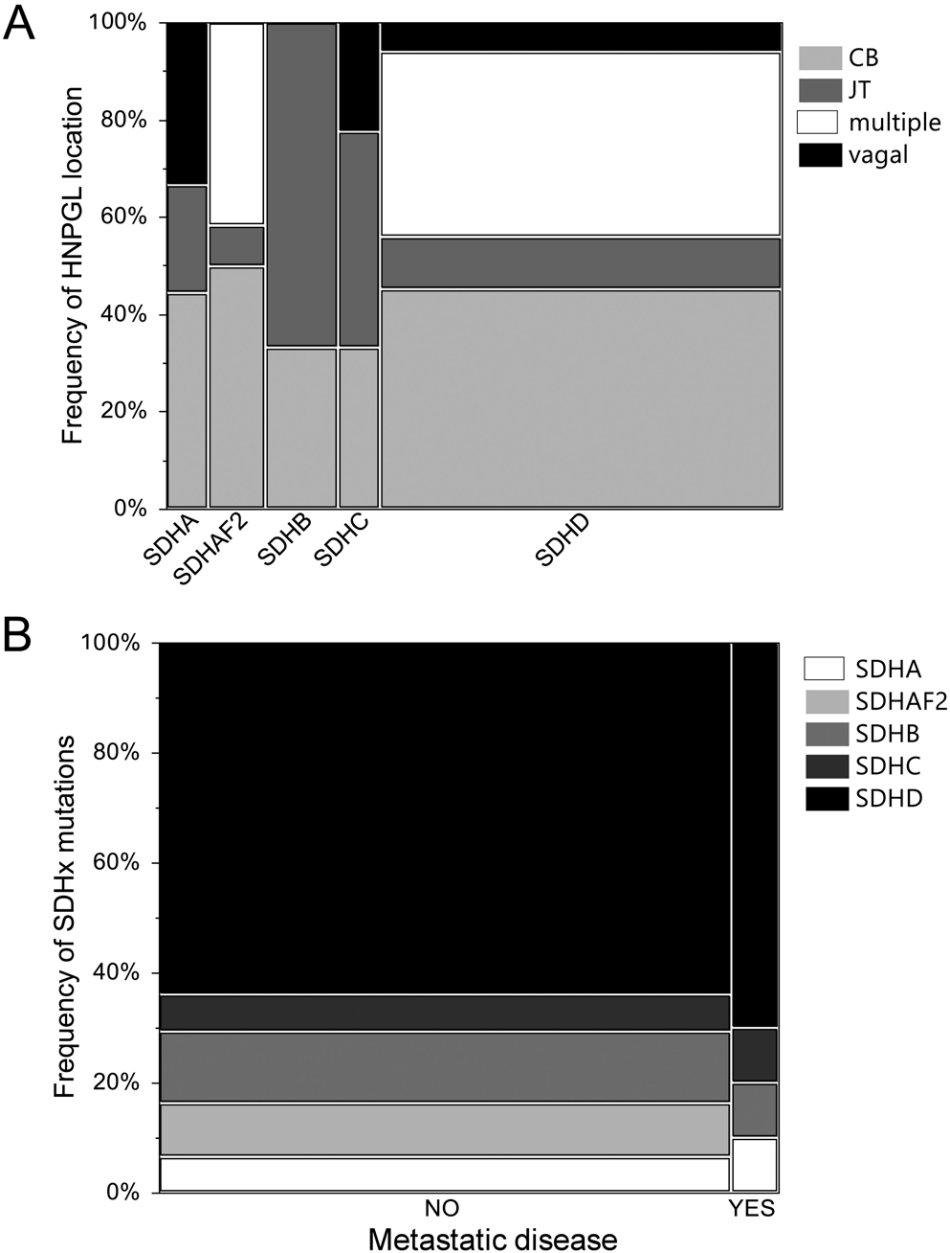
Frequency of mutations in *SDHx*genes in respect to HNPGL location (A) and occurrence of metastatic disease (B). Mosaic plots depict the width of the columns proportional to the number of patients in each group. (A) Mutations in the *SDHx* genes occur at different frequencies for various locations; Pearson’s chi-squared <0.0001. Especially, *SDHD* and *SDHAF2*mutations are associated with the occurrence of multiple HNPGLs. (B) No statistical difference was found between cases with and without metastatic disease in the number of patients with particular *SDHx* gene mutations; Pearson’s chi-squared = 0.8209. JT, jugulotympanic; CB, carotid body.

Germline mutations in *SDHx* genes were found to be in a higher percentage among patients with multiple or carotid body tumors, while less than one-third of jugulotympanic HNPGL patients had *SDHx* mutations (Table 3). Presentation of multiple HNPGLs was strongly associated with *SDHx* mutations and in particular, *SDHD* or *SDHAF2*mutations (Fig. 3A) in both men and women, but for all other locations, females less often had tumors due to *SDHx* mutations. Specifically, jugulotympanic HNPGLs in females were found in only 24% of cases associated with SDH loss compared to 55% of male cases. Overall, HNPGLs due to *SDHx* mutations were more common in males than females and were associated with an earlier diagnosis (Table 4).

Jugulotympanic HNPGLs were significantly smaller than all other types of HNPGL, while tumors with *SDHx* mutations were much larger at the time of resection (Tables 3 and 4). None of the jugulotympanic or vagal HNPGLs were bilateral in our cohort (Table 3). Bilateral presentation was more common in patients with germline *SDHx* mutations (Table 3). Metastatic disease occurred less often in patients with jugulotympanic HNPGLs and was not linked to *SDHx* mutation status in our cohort (Fig. 3B and Tables 3, 4). *SDHB* mutations were not associated with metastatic disease; among 17 *SDHB* mutation carriers, only one had metastases. In total, 18 patients suffered from metastatic disease; germline mutations in *SDHA*, *SDHB* and *SDHC* were present in one patient each, while *SDHD* mutations were found in seven patients (Fig. 3B and Table 2). For eight patients, no germline mutation was identified.

### Diagnostic implications of biochemical phenotypes in patients with HNPGL

**Figure 4:**
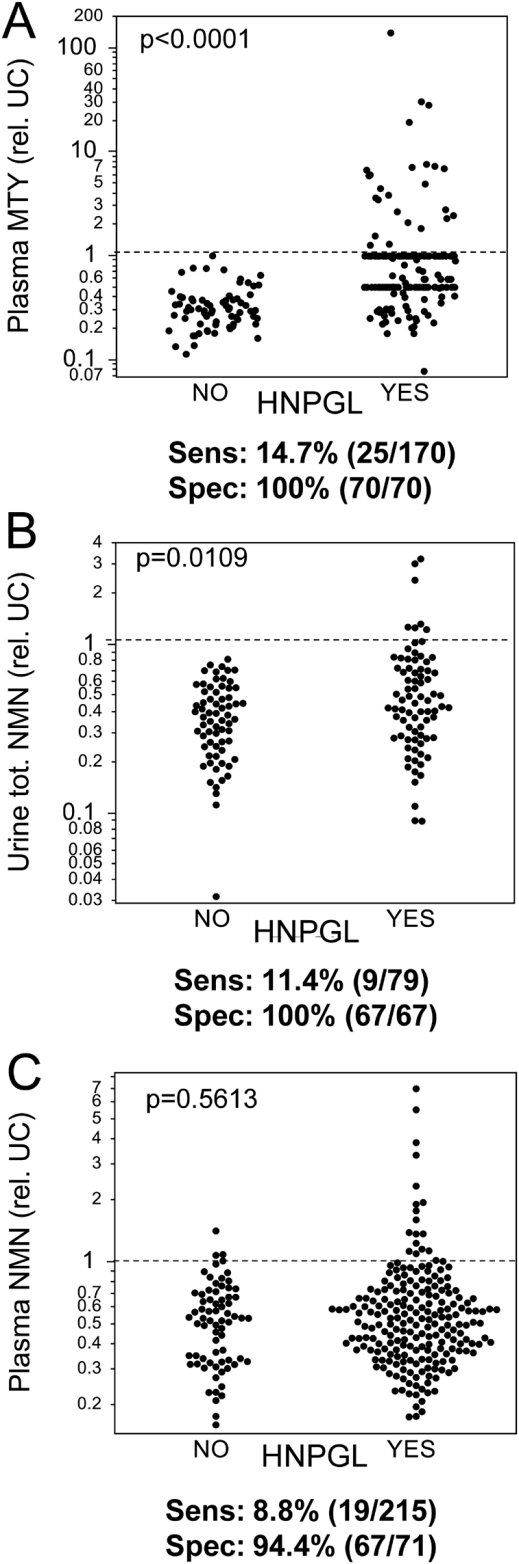
Plasma methoxytyramine (MTY, A), urinary total normetanephrine (NMN, B) and plasma NMN (C) relative to previously defined upper cut-offs. Displayed is the respective metabolite concentration in relation to upper cut-offs (UC, dashed line), defined based on age-specific plasma NMN and optimized for pheochromocytomas and non-HNPGLs (Eisenhofer *et al.* 2019). Significance was calculated by Wilcoxon rank-sum test comparing patients with and without HNPGLs. Sensitivity (Sens) and specificity (Spec) are displayed below the graphs.

In our cohort, 14.7 and 11.4% of patients with HNPGL had respective increases in plasma MTY and urine total NMN above previously defined cut-offs for these O-methylated metabolites (Eisenhofer *et al.* 2019) (Fig. 4A and B). Plasma NMN was not significantly different between patients with and without HNPGLs (Figs 2B and 4C), but 8.8% (19/215) had increases above age-specific upper cut-offs. However, among those 19 patients, eight were sampled in the seated position and had moderate increases of NMN, less than two-fold above age-specific upper cut-offs. Another three patients were sampled supine and had marginal increases in plasma NMN within the range of patients without disease and less than 1.5 times the upper cut-off. Finally, 3.7% (8/215) showed increases of more than 1.5-fold above upper cut-offs with adequate sampling procedures. Similarly, 3.7% (3/79) of patients had strong elevations of urinary total NMN. No patient with HNPGL had increased plasma MN or urine free and total MN above cut-offs (data not shown).

**Figure 5:**
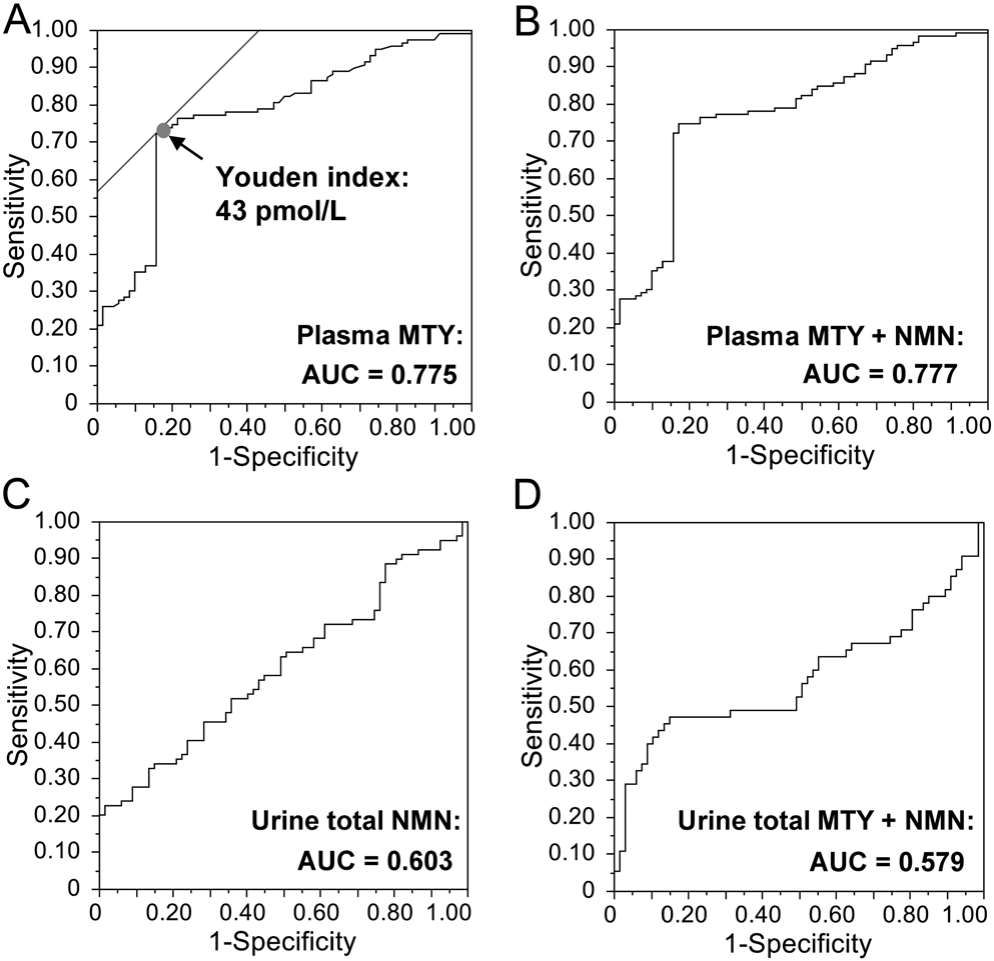
ROC curve analysis for plasma (A, B) and urine total (C, D) methoxytyramine (MTY) and normetanephrine (NMN). Plasma MTY alone (A) or the combination plasma NMN (B); *n*  = 189 (70 no-HNPGLs, 119 HNPGLs, excluding 51 patients, in whom plasma MTY was measured but only lower limit of quantification <100 pmol/L was given). Urine total NMN alone (C; *n*  = 146, 67 no-HNPGLs, 79 HNPGL) or in combination with urine total MTY (D; *n*  = 122, 67 no-HNPGLs, 55 HNPGL). AUC, area under the curve.

The routinely used upper cut-off for plasma MTY of 107 pmol/L was established from optimized 99.5% percentiles to ensure high diagnostic specificity among patients with catecholamine-producing chromaffin cell tumors that produced NMN as the main metabolite (Eisenhofer *et al.* 2019). At this cut-off, 21% (25/119) had positive test results. To establish more appropriate cut-offs for plasma-free MTY in patients with HNPGL, we employed two approaches: (1) use of 97.5 percentiles from our previously published reference population, which provided an upper cut-off of 60 pmol/L and (2) use of the Youden index according to ROC curve analysis (Fig. 5A). For the latter purpose, 51 Nijmegen patients were excluded due to a limit of quantification for MTY of 100 pmol/L for those measurements at that center. Lowering the upper cut-off from 107 to 60 pmol/L yielded a gain in diagnostic sensitivity to 30.3% (30/119) with a drop in specificity to 91.4% (64/70). Use of the Youden index indicated an optimal cut-off of 43 pmol/L with an associated diagnostic sensitivity of 73.9% (88/119) and specificity of 82.9% (58/70).

The area under the ROC curve (AUC) for plasma MTY was 0.775, while that for total urinary NMN was 0.603 (Fig. 5). According to AUCs, other plasma or urinary metabolites had negligible diagnostic utility both alone or in combination with plasma MTY or urinary deconjugated NMN. Combining plasma MTY and NMN resulted in an AUC similar to that of plasma MTY alone (Fig. 5B). Similarly, combining urine total NMN and MTY resulted in an AUC even lower than that of urinary deconjugated NMN alone (Fig. 5D).

## Discussion

Although our study did not establish associations of catecholamine-related biochemistry with tumor location or presence of *SDHx* mutations, we did establish numerous clinical features that differed according to tumor location and *SDHx* mutation status. Most importantly, we showed for the first time that jugulotympanic HNPGLs without *SDHx* mutations arise more often in older women and have distinct features compared to other HNPGLs. We also, for the first time, clarified the nature of catecholamine production in patients with HNPGLs compared to an appropriate group of patients without HNPGLs or any catecholamine-producing tumor.

It is well known that HNPGLs occur more frequently in women than men (Erickson *et al.* 2001, Smith *et al.* 2017, Rijken *et al.* 2019, Singh *et al.* 2019, Rana *et al.* 2021) and this was also reflected in our cohort. We systematically analyzed clinical features of HNPGLs and demonstrated that tumors of jugular and tympanic origin are more common in women (88%) than men. On the one hand, this compares with carotid body tumors at 62% in women, two-thirds of which were caused by germline *SDHx* mutations. On the other hand, jugulotympanic tumors in females are rarely associated with germline *SDHx* mutations, suggesting a distinct mechanism of tumorigenesis for jugulotympanic HNPGLs that are not due to *SDHx* mutations.

It was previously reported that tumor growth rates between HNPGLs of different locations were similar; however, a trend toward lower growth rates for jugulotympanic HNPGLs has been reported (Jansen *et al.* 2017). This agrees with our results identifying smaller sizes for jugulotympanic than other HNPGLs. Jansen *et al.* also identified a significant relationship between age of presentation and growth rate, with patients below 50 years having higher growth rates. In our cohort, the median age of first presentation for jugulotympanic HNPGLs was 54.5 years, while other HNPGLs presented at younger ages. This finding might be explained by the low percentage of *SDHx*-related syndromic cases within the group of jugulotympanic HNPGLs, since *SDHx*-mutated paragangliomas are known to present at relatively young ages (Eisenhofer *et al.* 2011, Jansen *et al.* 2017).

Previous studies in HNPGL patients showed metastatic disease to be predominantly associated with *SDHB* germline mutations (Boedeker *et al.* 2007, McCrary *et al.* 2019). Unexpectedly our findings were not consistent with these findings. Possibly, this discordance may relate to the care we took in excluding any patient with pheochromocytoma or paragangliomas at other locations, including patients with metastatic disease in whom the origins of the metastases could not be firmly attributed to an HNPGL.

Besides moderately lower plasma NMN in patients with carotid body tumors than other HNPGLs, there were no other significant differences in catecholamine-related biochemistry according to tumor location. This contrasts with a recent study that described carotid body and cervical sympathetic chain HNPGLs to be enriched with biochemically active tumors; nevertheless, in that study overall, only 9.2% of all patients had evidence of clinically significant tumoral catecholamine secretion (Smith *et al.* 2021) and also did not include measurements of plasma MTY or any group of patients without HNPGLs. These differences together with the fact that our cohort contained only one patient with a cervical sympathetic chain HNPGL may explain differences in our results vs that previous study.

In agreement with another study (van Duinen *et al.* 2013), plasma MTY was the best single parameter for the biochemical evaluation of HNPGLs. Adjusting the upper cut-off from the 99.5 percentile to the 97.5% percentile of a previously published reference population, that is 60 pmol/L (Eisenhofer *et al.* 2019), increased diagnostic sensitivity with a small loss in specificity. Calculations involving the Youden index suggest even further reduction of the upper cutoff with corresponding increases in sensitivity and decreases in specificity. Combining plasma NMN and MTY has increased sensitivity, which was also observed by van Duinen *et al*. (2013). However, by including patients without HNPGLs, we showed that the loss in specificity connected to the addition of plasma NMN abolished any overall diagnostic benefit. In agreement with current knowledge about HNPGLs, elevations in MN were not detected in any of the patients, while elevations in NMN occurred in only a small number of cases. Hence, we conclude that measurements of plasma MN and NMN have no relevance as diagnostic biomarkers in HNPGLs; only MTY may be useful for diagnosis. Nevertheless, measurements of plasma NMN serve some utility for identifying occasional patients with HNPGLs that produce norepinephrine and in whom preoperative management with alpha-adrenoceptor blockade may be indicated. For patients with HNPGLs that produce only dopamine, management with alpha-adrenoceptor blockade is unlikely to be useful.

Minimal utility of urine measurements of metanephrines for assessing biochemically active HNPGLs is also in agreement with previous studies (Erickson *et al.* 2001, van Duinen *et al.* 2010, 2013). However, those previous studies did not include patients without HNPGLs. With the inclusion of such patients in the present study, we established, based on ROC curve analysis, that compared to plasma-free MTY, the diagnostic utility of urine-free or total MTY is negligible. This is also in agreement with emerging concepts about different sources of plasma and urinary MTY, as well as previous findings that measurements of MTY in urine provide an insensitive method for assessing dopamine production by chromaffin cell tumors (Eisenhofer *et al.* 2018).

Based on measurements of plasma MTY, at least a third of HNPGLs in our cohort were identified as biochemically active, which is similar to previous findings (van Duinen *et al.* 2013, Smith *et al.* 2021). However, most current methods of analysis do not reliably measure MTY at the concentration range necessary to establish the production of the metabolite by small tumors typical of HNPGLs (Peitzsch *et al.* 2021). An exception might be the recently described LC-MS/MS method described by van Faassen *et al.* by which the use of *in situ* derivatization enables highly sensitive measurements of plasma MTY and other metabolites in as little as 50 µL of plasma (van Faassen *et al.* 2020). With such methods it should become possible to measure the production of MTY more precisely in small HNPGLs and thereby establish the true prevalence of biochemically active HNPGLs; as outlined here, this may reach 74%. Such a prevalence is supported by immunohistological examinations of HNPGL tissue, showing that the expression of the dopamine-producing enzyme, L-aromatic amino acid decarboxylase, is present in all samples (Osinga *et al.* 2015).

A limitation of the present study is that the analysis was carried out with a combination of prospectively and retrospectively included patients, among whom the number of prospective patients amounted to only 55 individuals with HNPGL. Furthermore, measurements of plasma and urine-free and total deconjugated metanephrines were not available for all patients. Another limitation concerns pre-analytical conditions in one of the centers. From 119 patients with measurements of plasma MTY, 48% were not fasted prior to blood withdrawal. Additionally, 34% of patients were not sampled in the supine position. We previously showed that the lack of fasting and supine sampling increases plasma NMN and MTY by up to two-fold in patients without catecholamine-producing tumors (Darr *et al.* 2014). Nevertheless, the comprehensive collection of other data together with the overall large number of patients with these rare tumors and the inclusion of an appropriate group of patients without disease represent strengths that set our study apart from previous work on catecholamine-related biochemistry of HNPGLs.

In conclusion, we clarify that tumor location and *SDHx* status do not determine biochemical activity of HNPGLs; rather tumor size appears to be a factor. About one-third of HNPGLs can be diagnosed biochemically using plasma MTY; however, due to limitations of analytical sensitivity, this proportion is likely underestimated. With the availability of more sensitive analytical methods, biochemical screening for HNPGLs may become more relevant and the true prevalence of dopamine-producing HNPGLs will be established. Additionally, we showed that the female predominance for HNPGLs is largely caused by a higher number of jugulotympanic HNPGLs in women and that these tumors are mostly unrelated to *SDHx* syndromes, rarely bilateral, typically smaller than other HNPGLs and less often result in metastases.

## Declaration of interest

The authors declare that there is no conflict of interest that could be perceived as prejudicing the impartiality of the research reported.

## Funding

This study was supported by the Deutsche Forschungsgemeinschaft http://dx.doi.org/10.13039/501100001659 (DFG, German Research Foundation) project number: 314061271 – TRR 205, the AES PI17/01796, co-financed by Fondo Europeo de Desarrollo Regional (FEDER), the European Union Seventh Framework Programme http://dx.doi.org/10.13039/100011102 (FP7/2007-2013) under grant agreement no. 259735, the Paradifference foundation and the Intramural Research Program of the NIH, NICHD.
